# Angularly anisotropic tunability of upconversion luminescence by tuning plasmonic local-field responses in gold nanorods antennae with different configurations

**DOI:** 10.1515/nanoph-2022-0037

**Published:** 2022-04-04

**Authors:** Chengda Pan, Qiang Ma, Shikang Liu, Yingxian Xue, Zhiyun Fang, Shiyu Zhang, Mengyao Qin, E Wu, Botao Wu

**Affiliations:** State Key Laboratory of Precision Spectroscopy, East China Normal University, Shanghai, People’s Republic of China; Joint Institute of Advanced Science and Technology, East China Normal University, Shanghai, People’s Republic of China; Chongqing Key Laboratory of Precision Optics, Chongqing Institute of East China Normal University, Chongqing, People’s Republic of China; Collaborative Innovation Center of Extreme Optics, Shanxi University, Taiyuan, Shanxi, People’s Republic of China

**Keywords:** Au nanorods, polarization, purcell effect, surface plasmon, upconversion luminescence

## Abstract

Optical polarization has attracted considerable research attention by extra detection dimension in angular space, flourishing modern optoelectronic applications. Nonetheless, purposive polarization controlling at nanoscales and even at the single-particle level constitutes a challenge. Plasmonic nanoantenna opens up new perspectives in polarization state modification. Herein, we report angular-dependent upconversion luminescence (UCL) of rare-earth ions doped upconversion nanoparticles (UCNPs) in both emission and excitation polarization via constructing angularly anisotropic plasmonic local-field distributions in gold nanorods (Au NRs) antennae with different configurations at a single-particle level. The UCL of UCNP tailored by plasmonic Au NRs nanoantennae is enhanced and exhibits linear polarization. The highest enhancement factor of 138 is obtained in the collinear Au NR-UCNP-Au NR configuration under parallel excitation. Simultaneously, the maximum degree of linear polarization (DOLP) of UCL with factors of 85% and 81% are achieved in the same structure in emission and excitation polarization measurements, respectively. The observed linear polarizations and UCL enhancements are due to varied resonant responses at 660 nm and the anisotropic near-field enhancement in different nanoantennae-load UCNP. The theoretical simulations reveal the periodic changing of near-field enhancement factors of nanoantennae in angular space with the incident light polarization angles and are well-matched with the experimental results.

## 1 Introduction

Polarization has been intriguing great attention on expanding a method for transmitting and distinguishing the information of light beside the intensity and wavelength [1, 2]. Its superiorities of angular-resolved features in space play a key role in modern optoelectronic applications, including holographic display and encryption [3], [4], [5], dynamic color tuning [6, 7], ultrasensitive photodetectors [8, 9], and anti-counterfeiting [10, 11]. Therefore, developing intrinsically polarized light-emitter or controllable artificial devices have been attracting numerous research interests during past decades [12], [13], [14]. Among the numerous fluorescent emitters, rare-earth ions doped upconversion nanoparticles (UCNPs) arouse tremendous scientific and industrial investigations owing to their excellent chemical and spectral properties, particularly to their unique nonlinear optical process. Upon sequential absorption of two or more photons, UCNPs can convert near-infrared or infrared radiation into visible luminescence [15], [16], [17]. Within different crystalline host matrix, the anisotropic optical properties of UCNPs are governed by the selection rules for the local site symmetries of rare-earth elements and also by the crystal symmetry [18], [19], [20], [21], [22]. The excitation direction along a particular crystallographic optical orientation in individual particle could obtain a strongly polarized luminescence. Besides, increasing dopant concentration would helpful for getting a better excitation polarization in an effective excitation configuration [21]. Hence, these intrinsically anisotropic optical properties of UCNPs have great potential applications for efficient orientation-sensing nanoprobes for bioimaging and diagnosis, polarization-sensitive photodetectors, and anticounterfeiting devices. However, achieving identical and controllable crystallographic optical orientation is still a hindrance for a variety of UCNPs, especially for sizes down to tens of nanometers. The small size and surface effects of nanoparticles would destroy the symmetric crystal field. Excavating another strategy for polarization state modification should be concerned.

Plasmonic nanoantennae have exceptional light-harvesting properties to confine light to local fields endowed by localized surface plasmon resonances (LSPRs) [23], [24], [25], [26]. The induced “hot spots” have intense local-field intensity with orders of magnitude more than the incident field, improving detection sensitivity and efficiency. Thanks to such exceeding properties, plasmonic nanoantenna has been extensively applied in nano-optical applications [27], [28], [29]. Constructing angular-resolved response based on plasmonic nanoantenna becomes a potential candidate for manipulating polarization states of light [13, 30, 31]. Specifically, the rod-shaped nanoantenna facilitates engineering light-emitting properties because of the in-coupling state in longitudinal mode and the out-coupling state in transverse mode [32], [33], [34], [35], [36]. There have been some works on plasmonic response polarization in different gold nanorods (Au NRs) configurations, showing amounts of fascinating phenomena and providing powerful tools for light manipulating and local-field modulation [36–38]. Through designing a configuration of quantum dot loaded asymmetric Au NRs, Xia et al. realized polarization-controlled Fano lineshapes [32]. Besides, Xue et al. demonstrated an enhanced polarized upconversion luminescence (UCL) by varying Au NRs diameter resulting from increased scattering cross-sections [35]. Consequently, incorporating plasmonic Au NRs nanoantenna with UCNPs can prospectively control not only intensity but also polarization state of emission by predesigned structures [35, 36, 39], [40], [41], [42], [43], [44]. However, a comprehensive investigation of UCL polarization state modification by Au NRs nanoantenna with different configurations, particularly at the single-particle level, is still lack of considerable concern.

Under this circumstance, we systematically presented angular-dependent UCL in emission and excitation polarization measurements based on different configurations by coupling a specific UCNP with Au NRs nanoantennae. A nearly spherical Yb^3+^, Er^3+^, and Mn^2+^ co-doped NaYF_4_ nanocrystal (NC) which shows isotropic UCL were precisely positioned in the hot spots of Au NRs nanoantenna with different configurations. The varied resonant responses at 660 nm and Purcell effect in coupled systems cause the different degrees of linear polarization (DOLP) in UCL at 660 nm. The maximum DOLP of UCL in emission and excitation polarization measurements were 85% and 81%, respectively. Meanwhile, significant UCL enhancements were obtained on different coupling configurations with a maximum enhancement factor of 138. Finally, we simulated local-field distributions of Au NRs nanoantenna with different configurations and theoretically analyzed their optical properties. The polarized emission tailored by plasmonic nanoantenna proposes a strategy for polarization state modification and is useful for polarization-based photodetection and imaging.

## 2 Materials and methods

### 2.1 Nanocrystal synthesis

Au NRs were synthesized by the colloid seed growth method according to previous report [45]. The NaYF_4_: Yb^3+^/Er^3+^/Mn^2+^@SiO_2_ UCNPs were purchased from Hefei Fluonano Biotech Co., Ltd, China.

### 2.2 Sample fabrication

UCNPs dispersed in absolute ethyl alcohol were first transferred to deionized water with concentration of 20 μg/mL, and then spin-coated on a clean glass substrate at speed of 2000 rpm/s. The average distribution density of UCNPs was 0.05 μm^−2^. The diluted aqueous solution of Au NRs with concentration of 4 μg/mL was next spin-coated on this glass substrate using same speed and dried in vacuum with an average distribution density of about ∼0.2 μm^−2^. The nanomanipulation technique for assembling Au NRs and UCNPs was applied on an AFM (Nanowizard II, JPK Instruments) by manipulation mode with setpoint of 0.2 V and velocity of 1 μm/s. The selected Au NRs and single UCNP are optically positioned with respect to the markers on the substrate, which facilitates AFM localization. Next, the selected UCNP and Au NRs were controlled to move together, and the spatial distance and adjacent angle between rods were adjusted with AFM tip.

### 2.3 Structural and optical characterization

TEM images were taken on a JEM-2100F microscopy operating at 120 KV. SEM images were pictured on a Sigma 300 (ZEISS) at 10 KV. AFM images were acquired using tapping mode with 1 Hz scanning speed and a resolution of 2 nm/pixel. Optical absorption spectra were obtained by using a UV–vis absorption spectrophotometer. UCL properties of the single UCNP and series nanoantennae-load UCNP were investigated in a combined system of a scanning confocal microscope and an AFM. As depicted schematically in Figure S3 (Supplementary Information), a continuous-wave laser at 980 nm was used as the excitation source. The controllable polarization direction of excitation was operated by rotating a half-wave plate after a Glan–Taylor prism at the output of the linear polarized laser. The modified laser was reflected by a dichroic mirror and focused by an oil-immersion microscope objective (×100, N.A. = 1.3, UPlanFLN, Olympus) with a focal spot size of ∼0.9 μm. The UCL spectra were collected by the same microscope objective and were spatially filtered by a pinhole with a diameter of 50 μm in a telescope system. After removing residual excitation laser signal using a band-pass filter covering 650 nm with a bandwidth of 40 nm or a short-pass filter cutting off at 780 nm, the UCL intensities were selectively detected by a single-photon detector based on a silicon avalanche photodiode (APD) for photon counting analysis or a spectrometer (SpectraPro-300i, Acton Research Corporation) for spectrum analysis. For time-resolved measurement, the excitation laser was chopped with a repetition rate of 600 Hz by a mechanical chopper (Terahertz Technologies C995-OH-3). The effective rise and decay lifetime data were recorded by the photons arriving at the ascending and falling edges of the pulse laser.

### 2.4 Numerical simulation

Numerical simulations were performed by using a commercial FDTD software (FDTD Solutions, Lumerical Solution, Inc. Canada). The Au NRs were modelled by average sizes with a radius of 20 nm, radius ends of 13 nm and length of 96 nm. The optical constants of gold were experimental data previously reported by Johnson and Christy [46]. Regarding the dielectric property, the silica-encapsulated UCNP was modelled as a homogeneous and isotropic silica sphere with a diameter of 45 nm. The refractive index of the surrounding medium was set to be 1.0. Au NRs-UCNP coupled systems on a thick substrate with a refractive index of 1.514 were illuminated by a total-field scattered field (TFSF) plane-wave source ranging from 400 to 1000 nm with discrete polarization angle from 0° to 90°. A 3D nonuniform meshing with a grid size of 1 nm was applied in the total field domain, with perfectly matched layer (PML) absorption boundary conditions. The total and scattered field power monitor is 260 nm and 300 nm away from the configurations, respectively. The TFSF source is in the middle of the total and scattered field power monitors. The scattering cross-section was estimated by calculating the summation of net power flowing into the total and scattered field simulation domains in a set of power monitors. To evaluate the electric field enhancement maps and the angular polarizations at 660 and 980 nm, the 2D frequency domain field profile monitors positioned in the half-height of nanoantennae were used. The angular polarizations were estimated by extracting the maximum values of electric field enhancement maps in corresponding polarization angles in the core of UCNP with a diameter of 31 nm. For the emission enhancement simulation, a dipole source is located in the center of silica sphere under polarization angle of 0°. A quantum efficiency analysis group is 300 nm away from the configurations to obtain radiative and nonradiative rates.

## 3 Results and discussion

**Figure 1:**
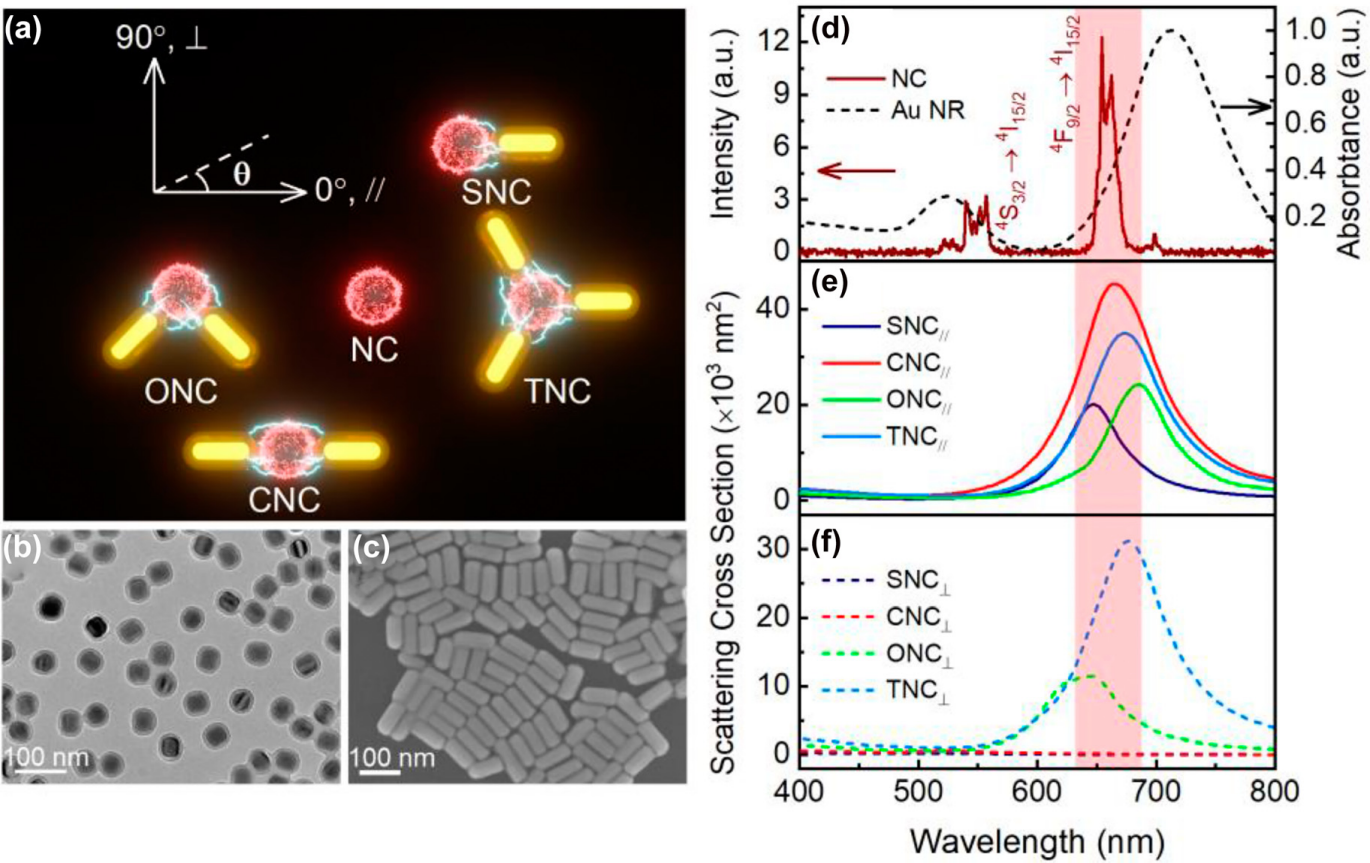
Schemetic of nanoantennae-load UCNP configurations, morphological characters of nanoparticles and simulated scatterring cross section. (a) Schematic of different nanoantennae-load UCNP configurations excited by an infrared laser beam with controlled polarization. (b) TEM image of Yb^3+^/Er^3+^/Mn^2+^ co-doped NaYF_4_ core–shell nanocrystals with the diameter of 45 nm and the silica shell thickness of 7 nm. (c) SEM image of Au NRs with the diameter of 40 nm and average length of 96 nm. (d) Upconversion luminescent spectrum of a single NC excited by 980 nm laser and absorption spectrum of Au NRs with the diameter of 40 nm in aqueous solution, where light red region represents the resonant region at 660 nm. (e, f) Simulated scattering spectra of different nanoantennae-load UCNP configurations in parallel (//, 0°) and perpendicular (⊥, 90°) with 0° axis marked in (a).

As illustrated in Figure 1(a), we propose series configurations of nanoantennae-load UCNP to produce angularly polarized excitation and emission in a specific UCNP at a single-particle level. The nanoantennae-load UCNP consists of one to three chemically synthesized colloidal Au NRs with varied coupling angles and a single UCNP. The configurations are assigned as single antenna loaded UCNP configuration (SNC), collinear dimer loaded UCNP configuration (CNC), orthogonal-angled dimer loaded UCNP configuration (ONC), and triangular-alike trimer loaded UCNP configuration (TNC) respectively. The UCNPs in our study are Yb^3+^, Er^3+^, and Mn^2+^ co-doped NaYF_4_ nanocrystals, which are further encapsulated with an amorphous silica shell. The dopant Mn^2+^ ions could reduce the transition possibilities between the excited states of green and red emissions of Er^3+^ by nonradiative energy transfer from the ^2^H_9/2_ and ^4^S_3/2_ levels of Er^3+^ to the ^4^T_1_ level of Mn^2+^, followed by back-energy transfer to the ^4^F_9/2_ level of Er^3+^, and enable us to quantitatively evaluate plasmonic enhancement effectiveness of different configurations at 660 nm [35, 47]. Figure 1(b) shows the representative transmission electron microscope (TEM) image of the core–shell UCNPs, which have a near-spherical shape with a diameter of about 45.2 ± 0.3 nm and a 7-nm-thick silica shell (Figure S1a, b). In previous reports, the optimized separation distance between UCNPs and Au nanoparticles was around 6–10 nm [35, 39]. The 7 nm-thick silica shell not only could prevent UCL quenching caused by Au NRs, but also enable Au nanorods to achieve high UCL enhancement. The UCL spectrum of the single UCNP depicted in Figure 1(d) (wine solid) exhibits an intense red emission peak at 660 nm and a weak green emission peak at 550 nm under the excitation of 980 nm laser. The red emission and green emission are attributed to the transitions of ^2^H_11/2_, ^4^S_3/2_ → ^4^I_15/2_ and ^4^F_9/2_ → ^4^I_15/2_ from Er^3+^ ions respectively [30, 35, 48]. See Figure S2b in Supplementary Information for more details about the energy level of UCNPs. The Au NRs were synthesized by colloid seed growth method [45] and their representative scanning electron microscope (SEM) image is shown in Figure 1(c). The average diameter of Au NRs is 39.8 ± 0.3 nm and the average length is 96.1 ± 0.4 nm (Figure S1c, d). Figure 1(d) shows the corresponding absorption spectrum in deionized water with LSPR position at about 708 nm (black short dash). Meanwhile, the simulated scattering spectra of different nanoantennae-load UCNP configurations under parallel (//, 0°) and perpendicular (⊥, 90°) polarized light using average sizes of Au NRs and UCNPs are shown in Figure 1(e) and (f). The plasmonic resonances of nanoantennae are blue-shifted due to the decreased refractive index of the medium surrounding Au NRs changed from water to glass in the air. They are all well overlapped with a narrow emission band around 660 nm in parallel orientation. The SNC and CNC in perpendicular orientation are out of resonance, but TNC and ONC are still well matched with red emission band. The local-field response that is resonant in one polarization direction and nonresonant in the orthogonal polarization direction opens up perspectives to achieve controllable angular polarization in excitation [32]. The sophisticated local-field distributions on different configurations also enable us to manipulate UCL intensities in diverse predesigned plasmonic structures.

**Figure 2:**
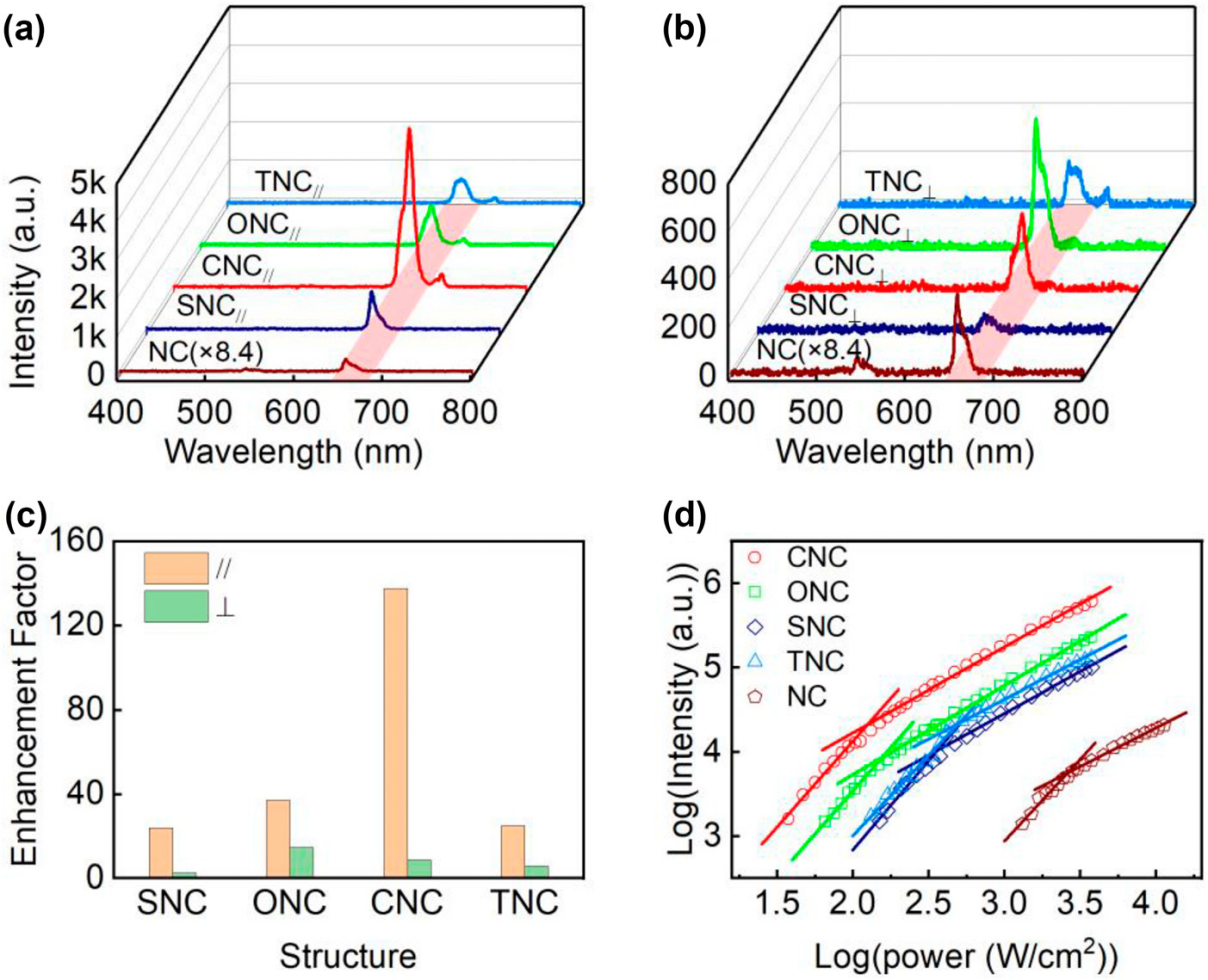
Experimental UCL spectra, enhacement factors and dependence of UCL intensities on laser power. (a, b) UCL spectra and (c) enhancement factor for NC and nanoantennae-load UCNP with different configurations under parallel (//, 0°) and perpendicular (⊥, 90°) polarized laser excitation with the excitation power density of about 7.53 × 10^2^ W cm^−2^. (d) UCL intensity dependent on the excitation power density for single NC and corresponding configurations of nanoantennae-load UCNP under parallel polarized laser excitation.

To investigate the influences of different nanoantenna configurations on the UCL characterizations of the UCNP, a homemade scanning confocal microscope combined with an atomic force microscope (AFM) depicted in Figure S3 (Supplementary Information) was used. Through AFM probe nanomanipulation technique, we construct Au NRs antennae loaded UCNP with different configurations and characterize their UCL properties *in situ* by emission and excitation polarization detection. The operating mode and parameters used in AFM nanomanipulation and topography are presented in Experimental Section. UCL spectra of series nanoantennae-load UCNP and the single UCNP under parallel and perpendicular polarized laser excitation are exhibited in Figure 2(a) and (b). Significant differences in UCL intensities from series nanoantennae-load UCNP are observed on these two orthogonal laser polarizations at an excitation power density of about 7.53 × 10^2^ W cm^−2^. Under parallel oriented excitation, the UCL intensities tailored by nanoantennae are dramatically amplified compared with the bare UCNP, and over two magnitudes are improved in CNC. While considering perpendicular-oriented excitation, UCL intensities of all nanoantennae-load UCNP are decreased in contrast to those under parallel orientation. The enhancement factors in Figure 2(c) were calculated to quantify UCL intensity before and after the UCNP tailored by nanoantennae. The original integrated intensities of UCL at 660 nm on the two orthogonal laser polarizations are illustrated in Figure S4. For the parallel polarized laser excitation, a noticeable enhancement by a factor of 138 is obtained in CNC, and the enhancement factors of 24, 37, and 25 are found in SNC, ONC, and TNC respectively. Although the UCL intensities are suppressed under perpendicular laser polarization against parallel orientation, they still display weak enhancement compared to the single bare UCNP. The enhancement factors are 2, 15, 9, 6 in SNC, ONC, CNC, and TNC, respectively. Such huge differences in enhancements convey the remarkable near-field interaction between Au NRs and UCNP in parallel excitation and weak coupling in perpendicular excitation. Thus, the following experimental description and analyse are focused on the UCL at the parallel excitation. Generally, UCL intensity has a nonlinear dependence on the excitation power density [49]:

(1) $$I_{\text{UCL}}\propto P^{n}$$

where *n* represents the number of excitation photons required to produce UCL. The photon number *n* involved in the energy transfer process can be determined by the slope of the double-logarithmic relation of UCL intensity versus excitation power density. Figure 2(d) plots the UCL intensities at 660 nm for the single UCNP and nanoantennae-loaded UCNP as a function of the laser power density colored in double-logarithmic relation with linear fittings. As depicted in Figure 2(d), at low excitation power density, the photon number *n* on NC, SNC, CNC, ONC, and TNC are 1.95, 2.12, 2.04, 2.05, and 1.91 respectively. And at high excitation power density, the photon number *n* on NC, SNC, CNC, ONC, and TNC are 0.92, 0.99, 1.02, 1.06, and 0.94, respectively. The UCL intensity typically exhibits quadratic dependence at low power density and linear dependence at high power density. Briefly, at low power density, the considerable linear decay of ^4^I_11/2_ → ^4^I_13/2_ leads to a two-step energy transfer process ^4^I_15/2_ → ^4^I_11/2_ and ^4^I_13/2_ → ^4^F_9/2_. While at high power density, the energy level of ^4^I_13/2_ is saturated and UCL is the dominant depletion mechanism, thus the slope of the red emission is close to 1 [50, 51]. Furthermore, the slope can be smaller than 1 at higher power density because of thermal quenching caused by the exposure of the 980 nm light [48, 50, 52, 53].

**Figure 3:**
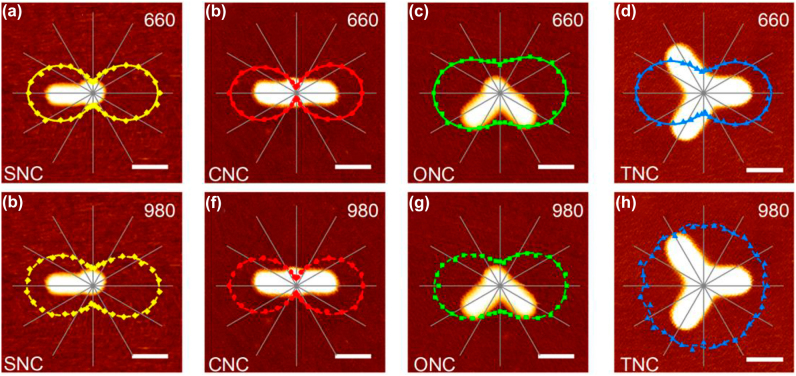
AFM topographic images of nanoantennae-load UCNP with different configurations and related polar plots of normalized UCL intensity at 660 nm (a–d) as a function of detection angle under excitation polarization angle at 0° and (e–h) as a function of excitation polarization angle under detection angle at parallel orientation. Scale bar: 100 nm. The solid curves are fitted by a sinusoidal function. The angle of ONC between two rods is 92° approximately, and the angles of TNC between the adjacent rods are about 123°, 114°, and 123° in clockwise from parallel orientation respectively.

We next quantify angular polarized responses of the UCNP before and after nanoantennae tailoring. The typical AFM topographic images of nanoantennae-load UCNP with different configurations are shown in Figure 3. Through the angular estimation between rods from images, the angle of ONC between two rods is 92° approximately, and the angles of TNC between the adjacent rods are about 123°, 114°, and 123° in clockwise from parallel orientation, respectively. The corresponding cross-section analyses are presented in Figure S5, which also clarify the highly uniform diameters of rods applied in measurements. At the meantime, the related angular-dependent polar plots of UCL intensities under different detection and excited orientations are depicted on the top of AFM topographic images. To analyze the sensitivity of the emission and excitation polarization, DOLP is used here [30]:

(2) $$\text{DOLP }=\left(I_{\text{max}}+I_{\text{min}}\right)\text{ / }\left(I_{\text{max}}+I_{\text{min}}\right)$$

where *I*_max_ and *I*_min_ represent the maximal and minimal UCL intensities from the polar plots. The UCL of near-spherical single UCNP shows little sensitivity to angular space (Figure S6) because of destroyed symmetric crystal field from varied crystal lattices orientation (Figure S2a). Under the parallel laser excitation (Figure 3(a)–(d)), the UCL intensities at 660 nm exhibit linear polarization with varied DOLP of 70%, 85%, 38%, and 53% in SNC, CNC, ONC, and TNC, respectively. When we fix the detection angle at parallel orientation and rotate the excitation polarization, comparing with their emission polarizations, the similar tendencies of UCL intensities are obtained in SNC, CNC, and ONC with corresponding DOLP of 57%, 81%, and 45% in Figure 3(e)–(g). However, TNC exhibits a nearly isotropic polarization as a function of excitation angle and its emission polarization appears a linear polarized trend with DOLP of 52%. The summarized DOLP is shown in Figure S4b. Generally, the significant angular responses of UCL in SNC and CNC are mainly owing to distinct resonant- and nonresonant-state alteration to emission processes (Figure 1(e) and (f)) over angle shifting, and Purcell effect from local-field enhancement in nanoantennae. For the nonresonant excitation under 980 nm, the obvious angular polarizations are mostly due to different absorption enhancements, which is originated from angular-depend nonresonant local-field enhancement from Au NRs. As for ONC case, the double resonances in parallel and perpendicular orientation depress the DOLP of UCL polarization, but angled nanoantenna still produces the angular polarization resulted from anisotropic spatial local-field distribution. The ideal configuration of TNC has three symmetry axes and rotated symmetry angles of 60°. Such highly symmetric structure shows highly symmetric local-field distribution and creates a nearly homogeneous excitation polarization in different angles. However, because of resonant peak overlapping between UCL and structure scattering at 660 nm, the emission polarization of TNC shows stronger dependence on local-field intensity comparing to its excitation polarization, and has a linear polarization under parallel excitation.

**Figure 4:**
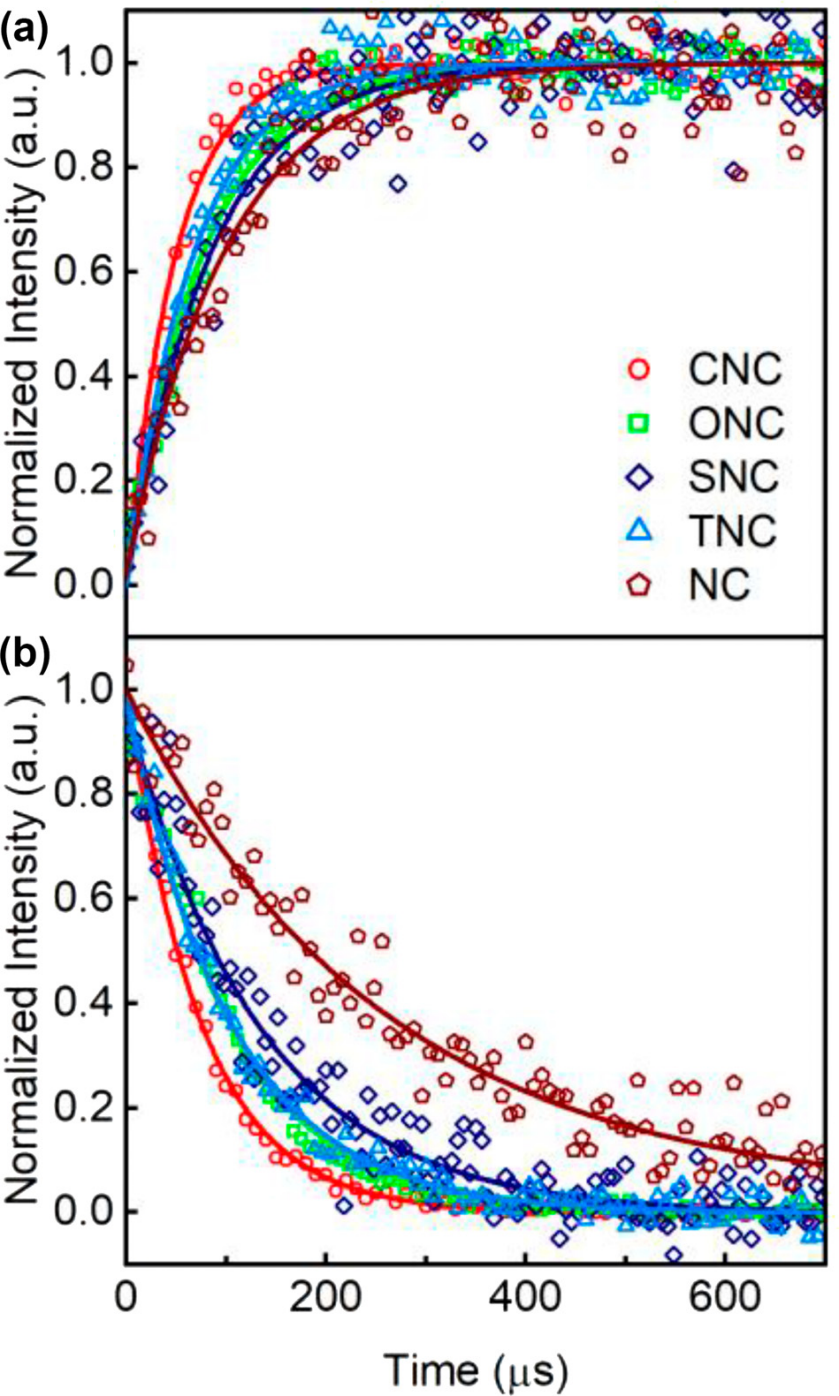
Experimental UCL rasing and decay times. (a) Rising and (b) decay times for the NC and different nanoantennae-load UCNP under parallel (//, 0°) polarized laser excitation.

To further understand the effect of different nanoantenna configurations on the upconversion process of UCNP, the time-resolved UCL measurements on the UCNP before and after assembling with Au NRs were performed. Figure 4 displays the lifetimes of emission and excitation for the NC and different nanoantennae-load UCNP under parallel excitation, and the data are listed in Table 1. The rise and decay times are both decreased after UCNP coupling with Au NRs nanoantennae. The configurations of SNC, ONC, and TNC have inconspicuous differences in lifetimes and CNC shows the shortest lifetimes. The decreased decay times of nanoantennae-load UCNP are generally caused by the following features: increased radiative rate of UCNPs, the energy transfer from UCNPs to metallic nanoantennae and the laser-induced local thermal effect of nanoantennae [35]. In our experimental measurements, the energy transfer and thermal effect could be nearly neglected because the SiO_2_ shell as the spatial spacer is about 7 nm in thickness and the excitation power density is 3.76 × 10^2^ W cm^−2^ which is lower than saturation excitation power. Therefore, the decreased decay times are mainly attributed to the increase of the radiative rate caused by enhanced local-field. Simultaneously, the rise times could be also influenced by the faster excitation rate from Yb^3+^ ions and internal energy transfer rate of Yb^3+^ to Er^3+^ ions in virtue of the nonresonant enhancement of the pump excitation field at 980 nm [35]. The time-resolved UCL spectra indicate that the UCL excitation and emission progresses are all accelerated.

**Table 1:** Rising and decay times for NC and different nanoantennae-load UCNP.

Sample	Rising times(μs)	Decay times(μs)
NC	98.3 ± 8.6	254.8 ± 18.2
SNC	83.7 ± 6.4	113.7 ± 6.4
ONC	78.4 ± 3.1	104.4 ± 2.3
CNC	49.6 ± 2.0	73.9 ± 1.0
TNC	66.9 ± 3.1	105.6 ± 2.9

**Figure 5:**
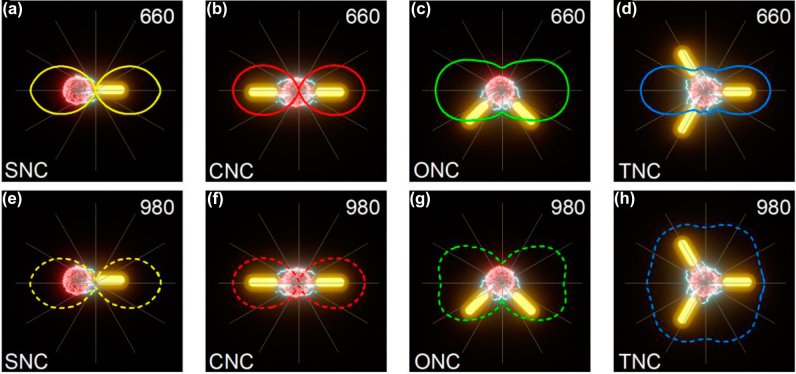
Schematics of nanoantennae-load UCNP with different configurations and corresponding simulated polar plots of normalized electric field enhancement |E|^2^/|E_0_|^2^ at 660 nm (a–d) and 980 nm (e–h) as a function of polarization angle. The solid curves are fitted by a sinusoidal function. The angles of TNC between the adjacent rods in (d) and (h) are about 123°, 114°, and 123° in clockwise from 0°, respectively.

To theoretically identify the experiment results, we performed theoretical simulations based on the finite-difference time-domain (FDTD) method using the average size of Au NRs and UCNPs. The normalized electric field enhancement polar plots of nanoantennae-load UCNP with different configurations in Figure 5 show exceedingly well match with the results in Figure 3. Those clear angular polarizations that occurred in SNC, CNC, and ONC reveal that the angular-dependent distribution of the local field in nanoantennae is a critical reason for linear polarization response of UCNP. As mentioned above, the angular field responses of TNC show sensitive feedback at 660 nm and inert feedback at 980 nm with symmetric breaking. In order to depict the impact of symmetric breaking, we simulated electric field enhancement polar plots of TNC with a perfect periodic angle of 120° (Figure S7a, b) and experimental angles of 123°, 114° and 123° (Figure 5(d) and (h)). As for excitation polarization of TNC in Figure S7b and Figure 5(h), both of them show low sensitivity in angular space and display nearly homogeneous local fields, which show great agreement with the result in Figure 3(h). Comparing with the experimental result of the UCL polar plot at 660 nm of TNC in Figure 3(d), the simulated local field of perfect angled TNC exhibits a flowery angular character in Figure S7a, while the small tilted TNC almost exhibit linear polarization and in consistent with experimental results. Obviously, this flowery polarization of the electric field at 660 nm is sensitive to structure symmetry, which has severe restrictions on the uniformity of rod length, spatial distance and adjacent angle of particles, and the absolutely coupling center of system. A slight symmetric breaking can collapse the homogeneous angular field response at 660 nm in TNC.

**Figure 6:**
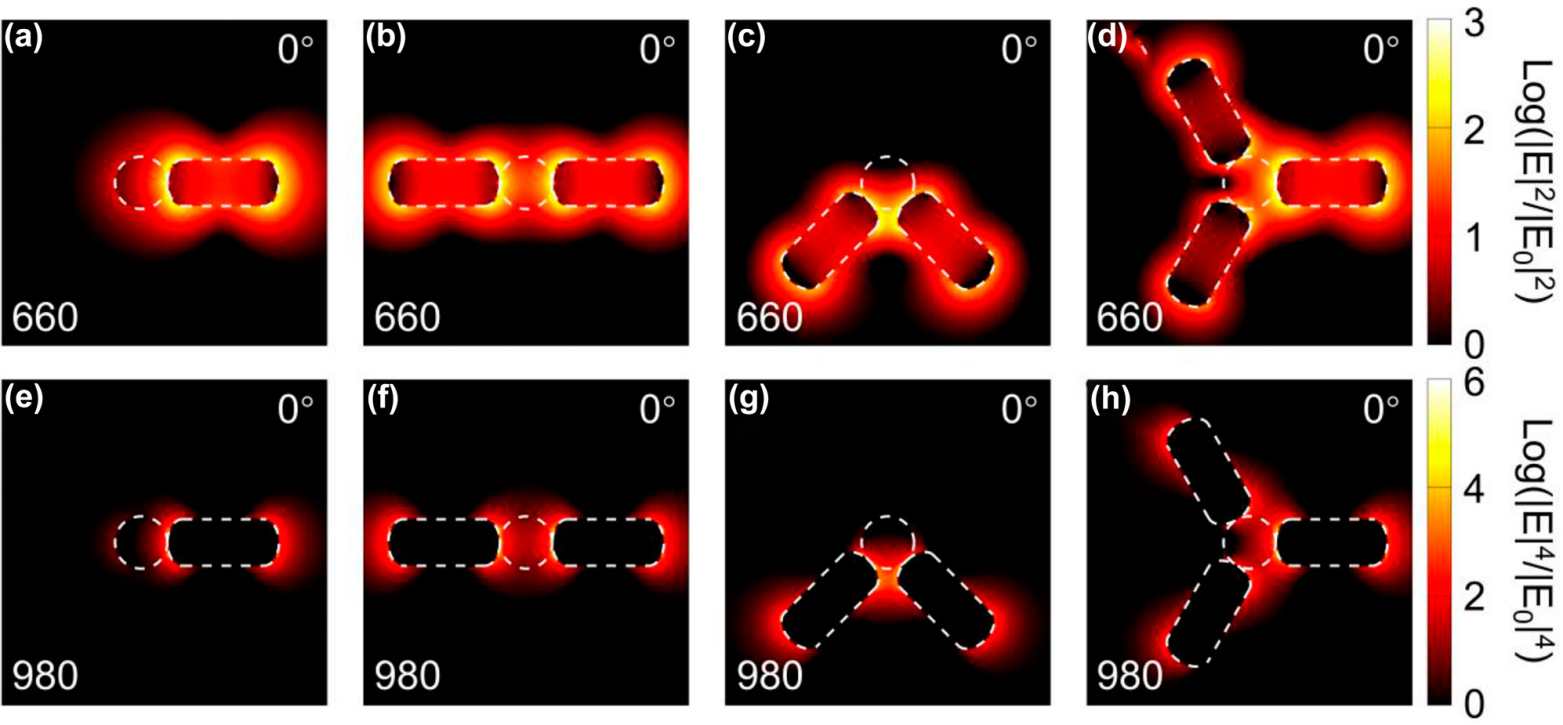
Simulated electric field enhancement distributions of nanoantennae-load UCNP with different configurations at (a–d) 660 nm and (e–h) 980 nm under polarization angle at 0°.

We also simulated the spatial distributions of electric field enhancement at 660 nm and 980 nm in parallel polarizations to get a better understanding of the LSPR effect in UCL enhancements. Figure 6 shows simulated LSPR near-field distributions of nanoantennae-load UCNP with different configurations illuminated at excitation polarization angle of 0°. The overall UCL enhancement factor is proportional to the product of emission and excitation enhancements [54, 55]. The emission enhancement depends on the competition between Purcell effect and antenna efficiency, which can be obtained by simulating the radiated and nonradiative rates of a dipole light source placed in the center of the UCNP under parallel polarization (See detailed information in Supplementary Information). Besides, the excitation enhancement is proportional to the local-field enhancement |E|^4^/|E_0_|^4^ accounted by a two-photon absorption process [56]. Table 2 shows the calculated emission and excitation enhancement factors at 660 and 980 nm and the overall UCL enhancement factor. CNC with the largest UCL enhancement with a factor of 5347 exhibits much stronger UCL enhancement than other Au NR configurations, which is consistent with the experimental result in Figure 2(c). It should be noted that the calculation of overall UCL enhancement is simplified, thus it is meaningless to directly compare the value of enhancement factors in Table 2 and Figure 2(c). But they still can indicate the coupling strength between UCNP and Au NR nanoantennae. In addition, TNC shows a little stronger UCL enhancement comparing with ONC, which is different from the experiment result. In fact, comparing the AFM cross-section analyses of TNC and ONC (Figure S5b), it can be seen that Au NRs and UCNP in TNC are not actually in complete contact indicated by a dip in the curve of AFM cross-section analysis of TNC, and the theoretical simulation assumes that they are in contact, which makes the simulation result larger. At the resonance wavelength of 660 nm, the “hotspots” induced by surface plasmon polaritons from the single rod on SNC and rod-rod coupling on CNC are both extremely localized on the ends of the rods (Figure 6(a) and (b)). The orthogonal angled dimer of ONC produces a new bonding mode along parallel orientation [36] and the “hot spot” is localized in the gap region (Figure 6(c)). In addition, the bonding mode of TNC has more complex mode coupling. It is obvious that, in rod-rod adjacent coupling in Figure 6(d), the horizon rod couples with its adjacent rods simultaneously. They produce two comparable bonding modes with equivalent energy due to the symmetry of TNC along the longitudinal axis of the horizon rod. The interaction from the top and bottom rods related to the horizon rod can be ignored on account of negligible weak coupling. When excited at 980 nm, although the excitation is in nonresonant wavelength, Au NRs have a little absorption at 980 nm and still produce a weak enhanced local field [43]. Such local field exhibits similar distributions with weaker coupling strength and distorted field morphologies compared with the status at 660 nm.

**Table 2:** Calculated emission and excitation enhancement factors and the resulted overall UCL enhancement factor of UCNP under parallel polarized laser excitation.

Sample	*F*_em_	*F*_ex_	UCL enhancement
SNC	10.1	41.0	414
ONC	10.6	115.6	1225
CNC	20.4	262.1	5347
TNC	14.1	146.4	2064

## 4 Conclusions

In summary, we demonstrate that the intrinsic unpolarized UCL of nearly spherical Yb^3+^, Er^3+^, and Mn^2+^ co-doped NaYF_4_ in angular space can be selectively modified through assembling with different configuration Au NRs antennae. The emission is preferred to emit towards the direction of largest electric field enhancement of Au NR antennae. Simultaneously, despite polarized excitation in parallel or perpendicular orientation, the UCL intensity of UCNP at 660 nm is obviously enhanced in all nanoantennae with a maximum enhancement factor up to ∼138 in CNC. Not only strengthened angular polarizations but also improved UCL intensities are mostly attributed to the following reasons: changed resonant interference between emission band at 660 nm from UCNP and plasmon resonances from different nanoantenna configurations, and Purcell effect from LSPR of nanoantenna at 660 nm. As for angular responses of the excitation at 980 nm, the angular anisotropic Purcell factors cause diverse polarizations. Finally, the FDTD simulations demonstrate near-field enhancements at both the pump wavelength and emission band and show great agreement with our results. The anisotropic UCL intensity of UCNP manipulated by different nanoantennae provides a strategy for polarization state modification and has great potential applications in dynamic color tuning, multicolor or holographic nanoscale light source, and polarization-based spectroscopy.

## Supplementary Material

See supplementary material for additional figures, including sizes distribution of particles; high-resolution TEM image and energy level of UCNPs; schematic of the experimental setup; summarized UCL intensities and DOLP in different configurations; AFM topographic images and cross-section analyses; AFM topographic images and polar plots of normalized UCL intensity of the UCNP; simulated polar plots of normalized electric field of TNC with perfect angle; and emission enhancement factor calculation.

## Supplementary Material

Supplementary Material Details
