# Supplementary material for: Angularly anisotropic tunability of upconversion luminescence by tuning plasmonic local-field responses in gold nanorods antennae with different configurations
Source: Nanophotonics. 2022 Apr 4;11(10):2349–59. doi: 10.1515/nanoph-2022-0037 (PMC11636477; doi:10.1515/nanoph-2022-0037)
Supplement: Supplementary file 1 — Supplementary Material Details [file j_nanoph-2022-0037_suppl.docx]

**Research Article - Supplementary Information**

Chengda Pan, Qiang Ma, Shikang Liu, Yingxian Xue, Zhiyun Fang, Shiyu Zhang, Mengyao Qin, E Wu and Botao Wu*

Angularly Anisotropic Tunability of Upconversion Luminescence by Tuning Plasmonic Local-field Responses in Gold Nanorods Antennae with Different Configurations

Supplementary information


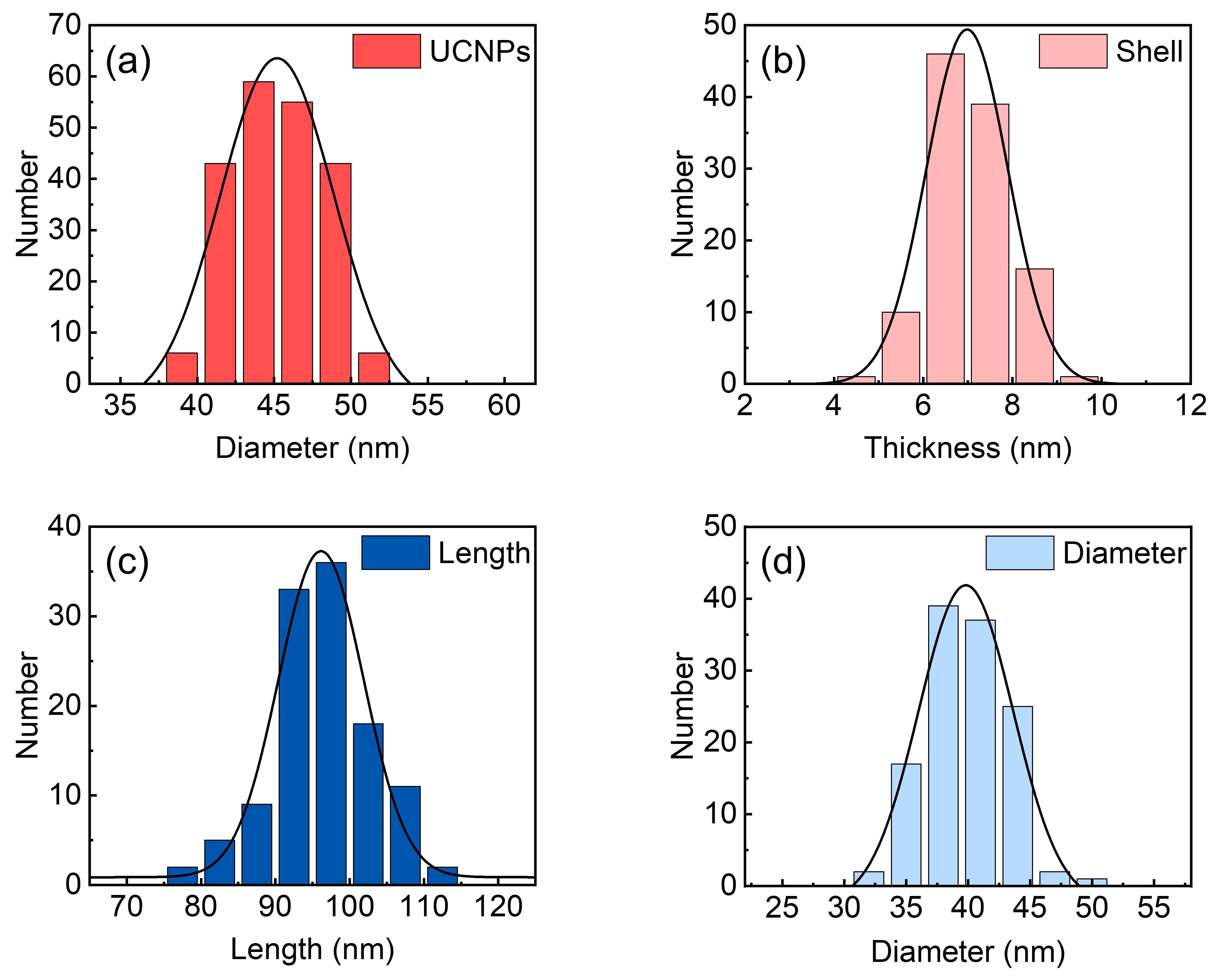


**Figure S1**. Size distributions of (a) silica-encapsulated UCNPs, (b) the thickness of silica shell, (c) the length and (d) diameters of gold nanorods. Mean sizes are 45.2 ± 0.3 nm, 7.0 ± 0.05 nm, 96.1 ± 0.4 nm and 39.8 ± 0.3 nm, respectively


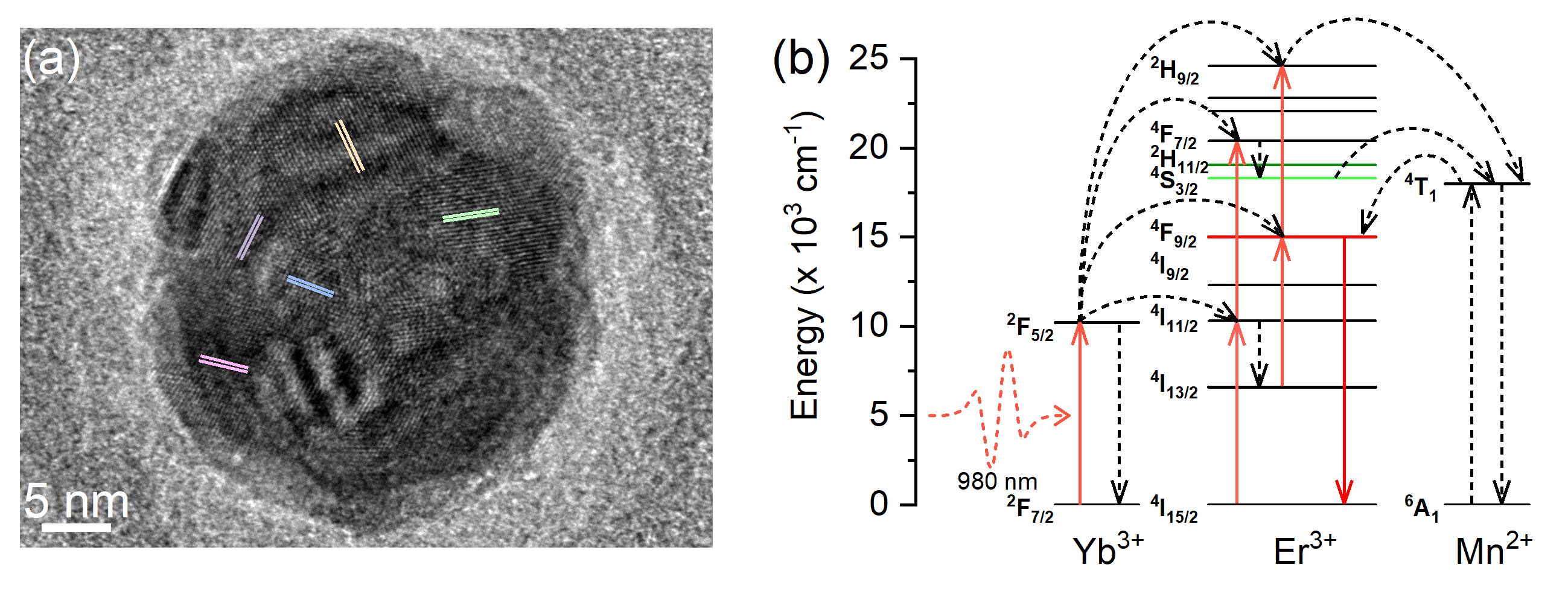


**Figure S2**. (a) High-resolution TEM image of the UCNPs with characteristic lattice plane information. (b) Energy level diagram with the possible upconversion transitions of Yb^3+^/Er^3+^/Mn^2+^ co-doped NaYF_4_ nanocrystals.


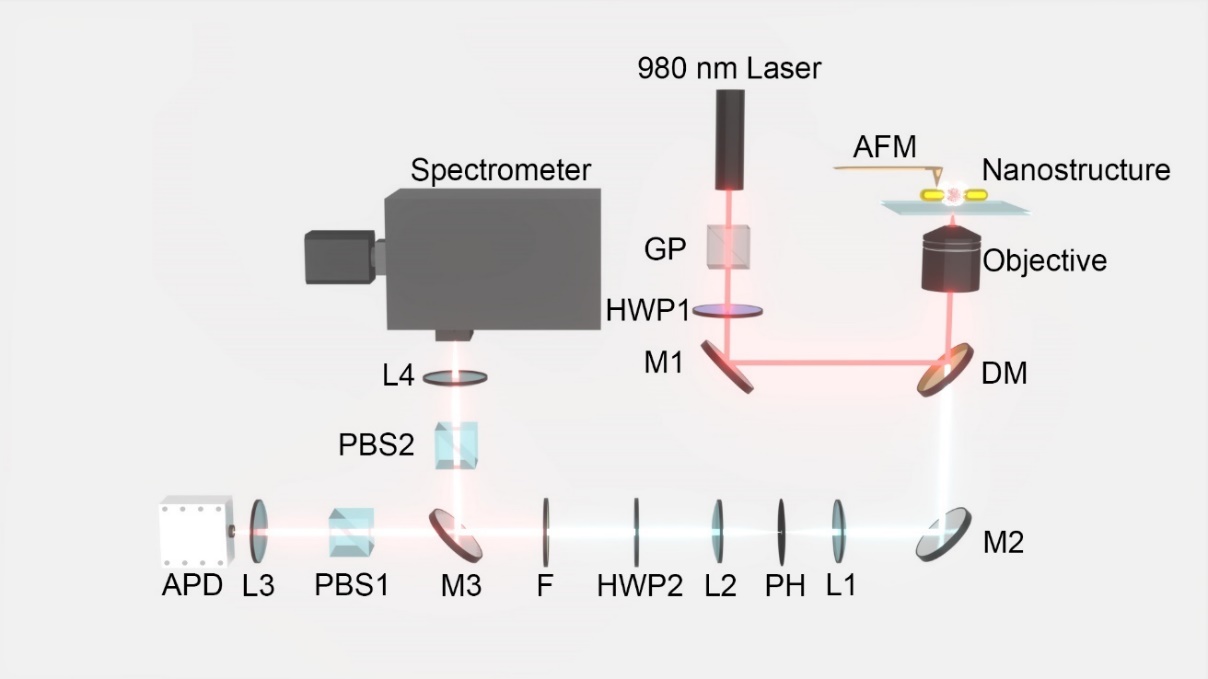


**Figure S3**. Schematics of experimental setup. An atomic force microscope assisted homemade scanning confocal microscope was used. AFM: atomic force microscope, GP: Glan prism, HWP: half-wave plate, M3: flip mirror, M: silver mirror, DM: dichroic mirror, L: lens, PH: pinhole, F: filter, PBS: polarized beam splitter, APD: single-photon detector based on Si-avalanche photodiode.


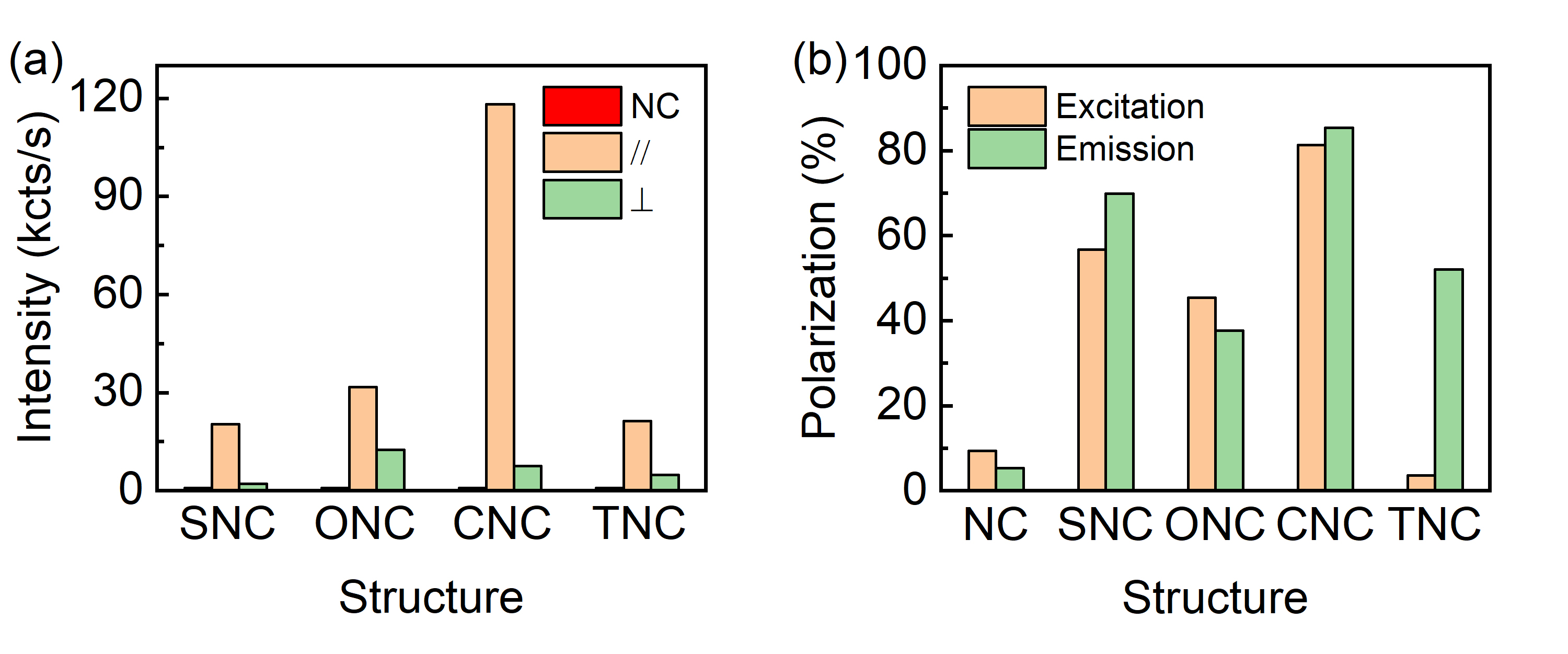


**Figure S4**. (a) UCL intensity comparison for the single UCNP before and after assembling with Au NRs under parallel (//, 0°) and perpendicular (⊥, 90°) polarized laser excitation. (b) DOLP of different nanoantennae-load UCNP for emission polarization under the excitation polarization angle at 0° (Emission) and excitation polarization as a function of excitation polarization angle (Excitation). The excitation power density is about 7.53 × 10^2^ W cm^-2^.


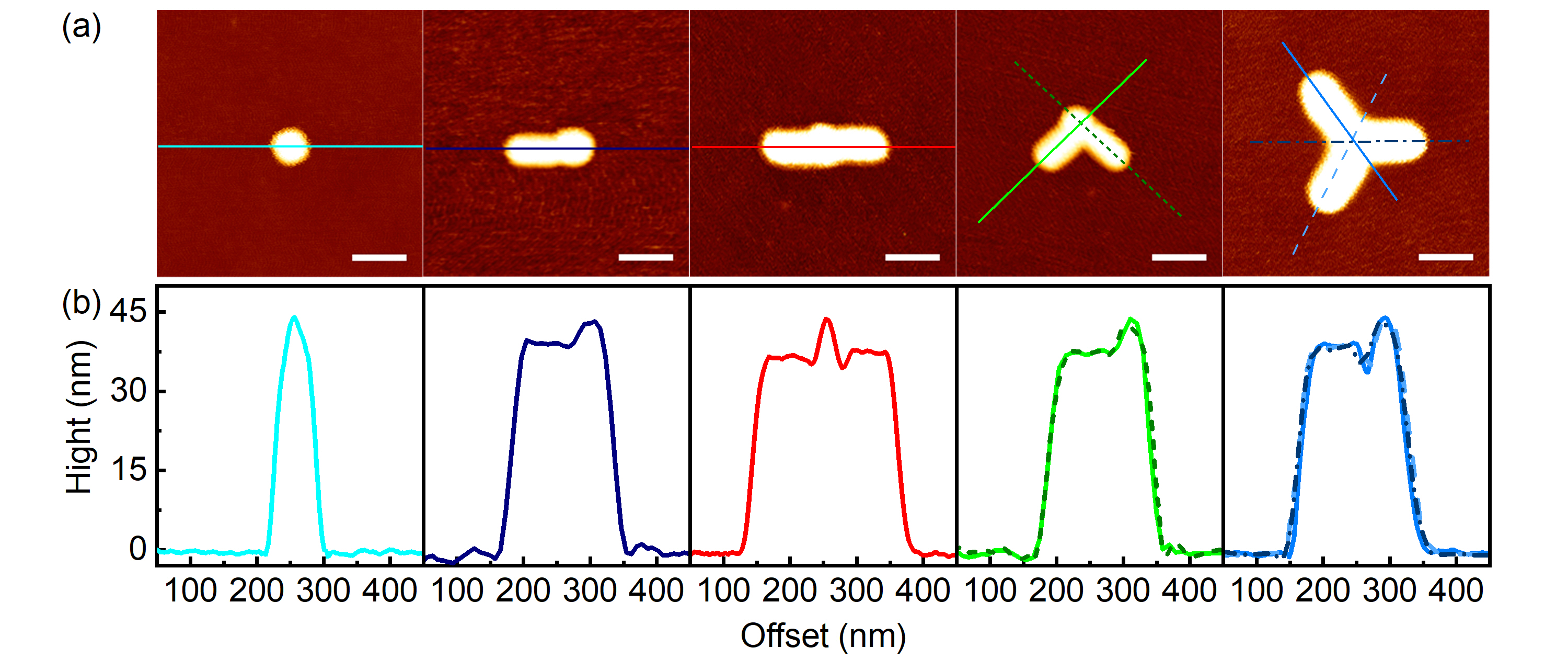


**Figure S5**. (a) AFM topographic images of the UCNP and different nanoantennae-load UCNP configurations. Scale bar: 100 nm. (b) Corresponding cross-section analyses along the axes direction of longitudinal of Au NRs.


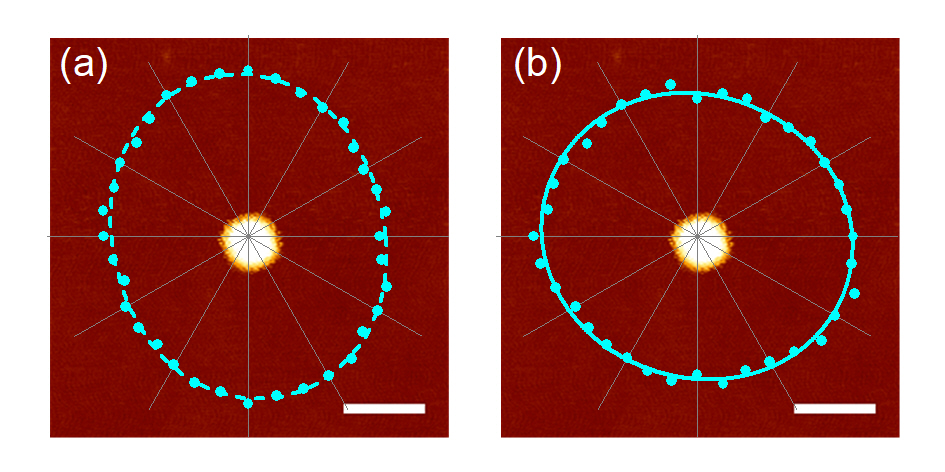


**Figure S6**. AFM topographic images of the UCNP and related polar plots of normalized UCL intensity at 660 nm (a) under excitation polarization angle at 0° and (b) as a function of excitation polarization angle. Scale bar: 100 nm.


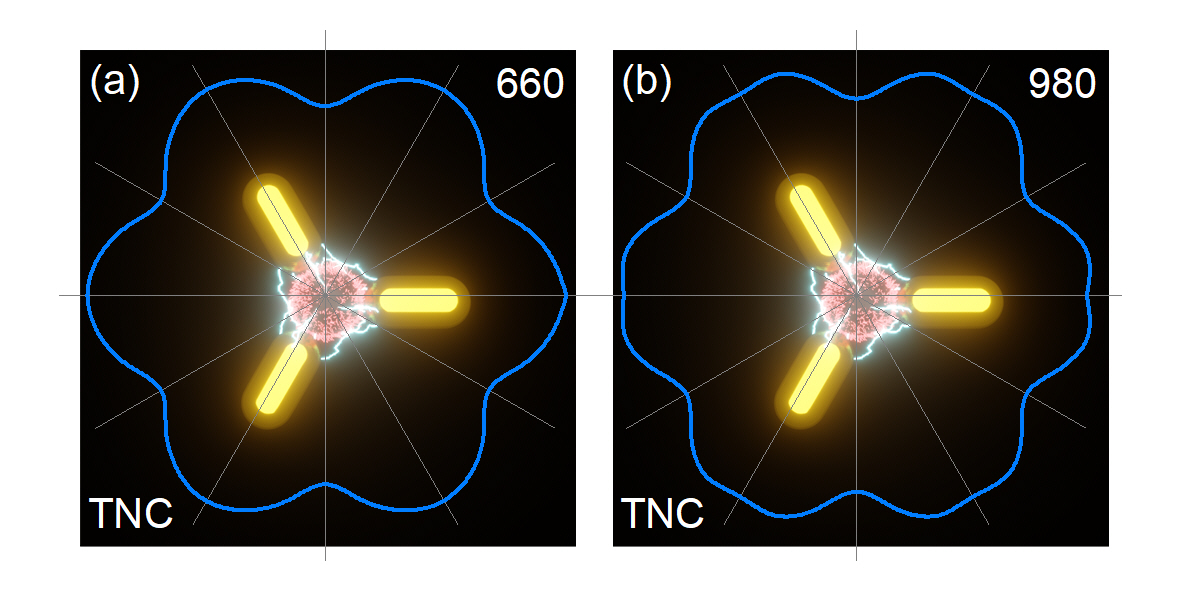


**Figure S7**. Schematics of TNC with the perfect angle and related polar plots of normalized electric field |E|^2^/|E_0_|^2^ at 660 nm (a) under excitation polarization angle at 0° and (b) as a function of excitation polarization angle. The solid curves are fitted by a sinusoidal function.

Emission Enhancement Calculation

When an emitter is nearby a metallic nanostructure, the metallic nanostructure accelerates the radiative decay rate of the emitter and provides an additional pathway for energy dissipation. The ratio $\eta_{a}=\Gamma_{\mathrm{em}}^{R}/\left( \Gamma_{\mathrm{em}}^{R}+\Gamma_{\mathrm{em}}^{\mathrm{NR}} \right)$ is defined as the antenna efficiency of the metal nanostructure. $\Gamma_{\mathrm{em}}^{R}$ is the accelerated radiative decay rate of the emitter and $\Gamma_{\mathrm{em}}^{\mathrm{NR}}$ is the non-radiative decay rate due to energy dissipation. The Purcell factor $F$ can be expressed as the radiative decay rate enhancement. The emission enhancement of the UCNP can be expressed as [1]:

$$\begin{aligned} F_{\mathrm{em}}=\frac{1}{\left( 1-\eta_{0} \right)/F+\eta_{0}/\eta_{a}}, \end{aligned}$$

Here, the quantum efficiency $\eta_{0}$ of the UCNPs is assumed as 1% because of small absorption cross sections caused by the forbidden transitions between 4f orbitals of the lanthanide dopants [2].

**Table S1.** Purcell Factors and Antenna Efficiency Calculated by the simulated radiative and nonradiative decay rates.

| Sample | 660 nm | |
| --- | --- | --- |
|  | $\boldsymbol{F}$ | $\boldsymbol{\eta}_{\boldsymbol{a}}$ |
| SNC | 12.6 | 0.49 |
| ONC | 13.7 | 0.45 |
| CNC | 32.8 | 0.57 |
| TNC | 19.6 | 0.49 |

References

1. X. Liu and D. Y. Lei, “Simultaneous excitation and emission enhancements in upconversion luminescence using plasmonic double-resonant gold nanorods,” *Sci. Rep.*, vol. 5, p. 15235, 2015.
2. J. C. Boyer and F. C. J. M. V. Veggel, “Absolute quantum yield measurements of colloidal NaYF_4_: Er^3+^, Yb^3+^ upconverting nanoparticles,” *Nanoscale*, vol. 2, pp. 1417–1419, 2010.
